# TAK1 inhibitor NG25 enhances doxorubicin-mediated apoptosis in breast cancer cells

**DOI:** 10.1038/srep32737

**Published:** 2016-09-07

**Authors:** Zhenyu Wang, Huiyuan Zhang, Minghao Shi, Yang Yu, Hao Wang, Wen-Ming Cao, Yanling Zhao, Hong Zhang

**Affiliations:** 1 Department of Breast Surgery, the Second Hospital of Jilin University, Changchun, Jilin 130041, China; 2 Department of Pathology, University of Texas MD Anderson Cancer Center, Houston, Texas 77030, USA; 3 Department of Translational and Molecular Pathology, University of Texas MD Anderson Cancer Center, Houston, Texas 77030, USA; 4 Department of Blood Transfusion, the Second Hospital of Jilin University, Changchun, Jilin 130041, China; 5 Department of Hepatopancreatobiliary Surgery, the Second Affiliated Hospital of Harbin Medical University, Harbin, Heilongjiang 150086, China; 6 Department of Medical Oncology, Zhejiang Cancer Hospital, Hangzhou, Zhejiang 310022, China

## Abstract

Doxorubicin (Dox, Adriamycin) has been widely used in breast cancer treatment. But its severe cardio-toxic side effects limited the clinical use. Dox treatment can induce DNA damage and other accompanying effects in cancer cells, and subsequently activates nuclear factor κB (NF-κB) pathway which has a strong pro-survival role in different types of malignancy. We hypothesize that blocking NF-κB pathway may sensitize breast cancer cells to Dox chemotherapy. TGFβ-activated kinase-1 (TAK1) is a key intracellular molecule participating in genotoxic stresses-induced NF-κB activation. Targeting TAK1 as a strategy to enhance cancer treatment efficacy has been studied in several malignancies. We showed that NG25, a synthesized TAK1 inhibitor, greatly enhanced Dox treatment efficacy in a panel of breast cancer cell lines. In this pre-clinical study, we found that NG25 partially blocked Dox-induced p38 phosphorylation and IκBα degradation and enhanced Dox-induced cytotoxic effects and apoptosis in all breast cancer cell lines tested. Taken together, we provided clear evidence that NG25 sensitizes the breast cancer cells to Dox treatment *in vitro.* This combination may be an effective and feasible therapeutic option maximizing Dox efficacy and meanwhile minimizing Dox side effects in treating breast cancer.

Breast cancer is a group of heterogeneous diseases based on gene profiles, biological behaviors, morphological features and clinical outcomes^1^. It accounts for 22% of all female cancers and 26% in affluent countries, which makes it the most common carcinoma in women. It is estimated that about 6% of women develop invasive breast cancer before age 75 in affluent populations in North America, Europe and Australia^2^. With the improvement in the combination of more effective local regional disease control and systemic treatment protocols, the mortality of breast cancer has been reduced dramatically^3^. The successful cancer-specific targeted therapies involving the endocrine therapy on estrogen receptor(ER)-positive tumors and HER2-targeted therapy on HER2 over-express tumors are major contributors. However, the cytotoxic agents are still the mainstream medications on advance breast cancer, especially for triple negative tumors^4^. Doxorubicin (Dox, Adriamycin) is one of the most commonly used chemotherapy drugs for treating breast cancer. Dox-containing adjuvant chemotherapy is recommended as the first line anti-cancer drug in 2016 NCCN’s breast cancer guidelines, especially for HER-2 positive and triple negative breast cancer patients^5^. Dox is well known to cause dose-dependent and progressive cardiac damage^6^,^7^, profoundly affecting its clinical use. Different formulas of Dox such as liposomal doxorubicin or in combination with cardio-protective Dexrazoxane have been used clinically to minimize the cardio toxicity. These render limited benefit^8^,^9^,^10^. Identification of new ways to maximize the benefits of Dox and at the same time minimize cardiac damage is in need for better treatment outcome.

TAK1 was first discovered in 1995 as a mitogen-activated protein kinase kinase kinase (MAP3K), which is activated by TGF-β and bone morphogenetic protein (BMP)^11^. Recent studies emphasized that TAK1 has a powerful pro-survival role through activating the IκB kinase (IKK)-NF-κB pathway^12^. In addition, the activated TAK1 can phosphorylate and activate MAPKKs leading to activation of MAPKs such as ERK, p38 and JNK^13^. The activation of NF-κB and MAPKs induces downstream expression of inflammatory cytokines and anti-apoptotic proteins which can block apoptosis and promote cell proliferation^14^,^15^. Furthermore, elevated NF-κB activity has been shown to contribute to the development of resistance to chemotherapy, endocrine therapy and radiation therapy. Due to the critical role of TAK1 in cancer development and drug resistance, targeting TAK1 has been suggested as a promising anti-cancer strategy and have been studied in pancreatic cancer, breast cancer, colon cancer and non-small cell lung carcinoma^4^,^16^,^17^,^18^. Based on the knowledge that TAK1 mediates NF-κB activation in response to genotoxic stresses, 5Z-7-oxozeaenol (5Z-O), an irreversible ATP-competitive TAK-1 inhibitor, has been used to enhance the sensitivity of neuroblastoma cells to Dox therapeutic treatment^19^,^20^,^21^. Recently, 5Z-O also has been shown inhibiting TAK1 activity and then suppressing downstream signaling pathways including p38 and IκB kinase (IKK) pathways in breast cancer cells^22^. Furthermore, inhibition of TAK-1 activity by knocking out TAK1-aasociated molecule TAB1 significantly suppressed breast cancer growth and metastasis *in vivo*^22^. Considering the aforementioned information, we hypothesize that TAK1 inhibitor would probably enhance Dox-mediated cytotoxicity in breast cancer cells.

NG25 is a synthesized type II TAK1 inhibitor which binds to the ATP binding pocket of the target kinase^23^. Comparing to 5Z-O, a compound isolated from fungi, NG25 has economic benefits. Recently, NG25 has been shown to be able to effectively inhibit TAK-1 activity in several studies^24^,^25^,^26^. However, the potential therapeutic effect of NG25 on cancer treatment has not been tested. In this preclinical study, we tested the cytotoxic effect of NG25 and its effects on Dox treatment on breast cancer cells by using a panel of breast cancer cell lines including T-47D, MCF7, HCC1954, MDA-MB-231, and BT-549 (representing ER/PR+, HER2+, or triple negative, respectively).

## Results

### TAK1 inhibitor NG25 suppresses the viability and proliferation of breast cancer cells

**Figure 1:**
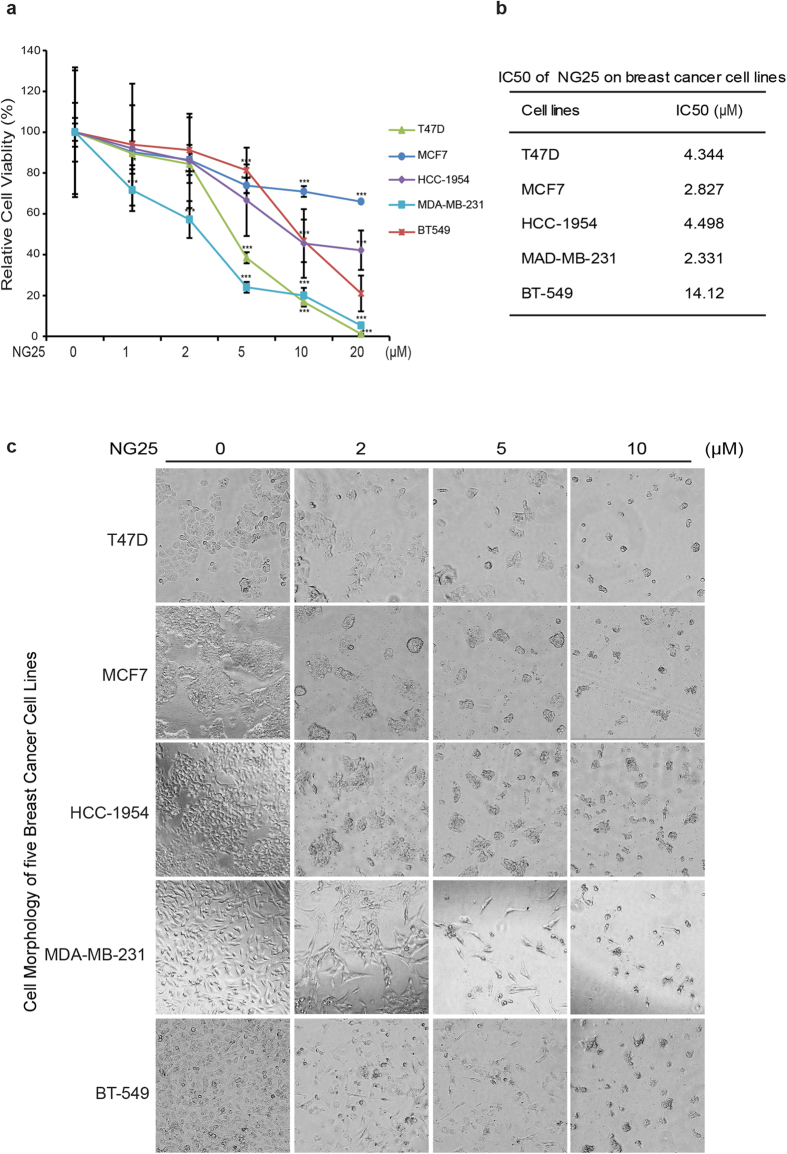
NG25 shows cytotoxic effect on breast cancer cells. (**a**) Cytotoxic effect of NG25 on breast cancer cells in CCK-8 assay. Five human breast cancer cell lines T-47D, MCF7, HCC1954, MDA-MB-231, and BT-549 were treated with NG25 at 0, 1 μM, 2 μM, 5 μM, 10 μM, and 20 μM for 72 h, then subjected to a CCK-8 assay. The absorbance of each well was measured at 450 nm and plotted for the cell viability curve. The data are represented as mean ± SD. *P* values < 0.01 (**) or < 0.001 (***) were indicated. (**b**) IC50 values of NG25 in breast cancer cell lines were listed. (**c**) Photographed cells of breast cancer cells treated with indicated concentrations of NG25 for 72 h, and cell morphology was captured using optical microscope.

In order to assess the inhibitory effect of NG25 on the proliferation of breast cancer cells, five breast cancer cell lines, including T-47D, MCF7, HCC1954, MDA-MB-231, and BT-549, were selected, representing major molecular subtypes of breast cancers (Table 1)^27^,^28^. All of the cells were treated with NG25 at the indicated concentrations from 1 μM to 20 μM for 72 h, and then applied to the Cell Counting Kit-8 (CCK-8) assay. The results showed that NG25 treatment reduced cell viability of all tested breast cancer cell lines in a dose dependent manner (Fig. 1a). The IC50 of NG25 on breast cancer cell lines were calculated and listed in Fig. 1b. Morphological changes pictures of the breast cancer cells after treatments for 72 h confirm the inhibitory effect of NG25 (Fig. 1c). The toxicity of NG25 is much lower to normal breast epithelial cell lines HMEC and MCF-12A compared to MDA-MB-231 cell line (Supplemental Fig. 1). These data demonstrate that the TAK1 inhibitor NG25 alone can suppress breast cancer cell proliferation, but the efficacy varied in different cell lines.

### NG25 enhances the cytotoxic effect of Dox on breast cancer cells

**Figure 2:**
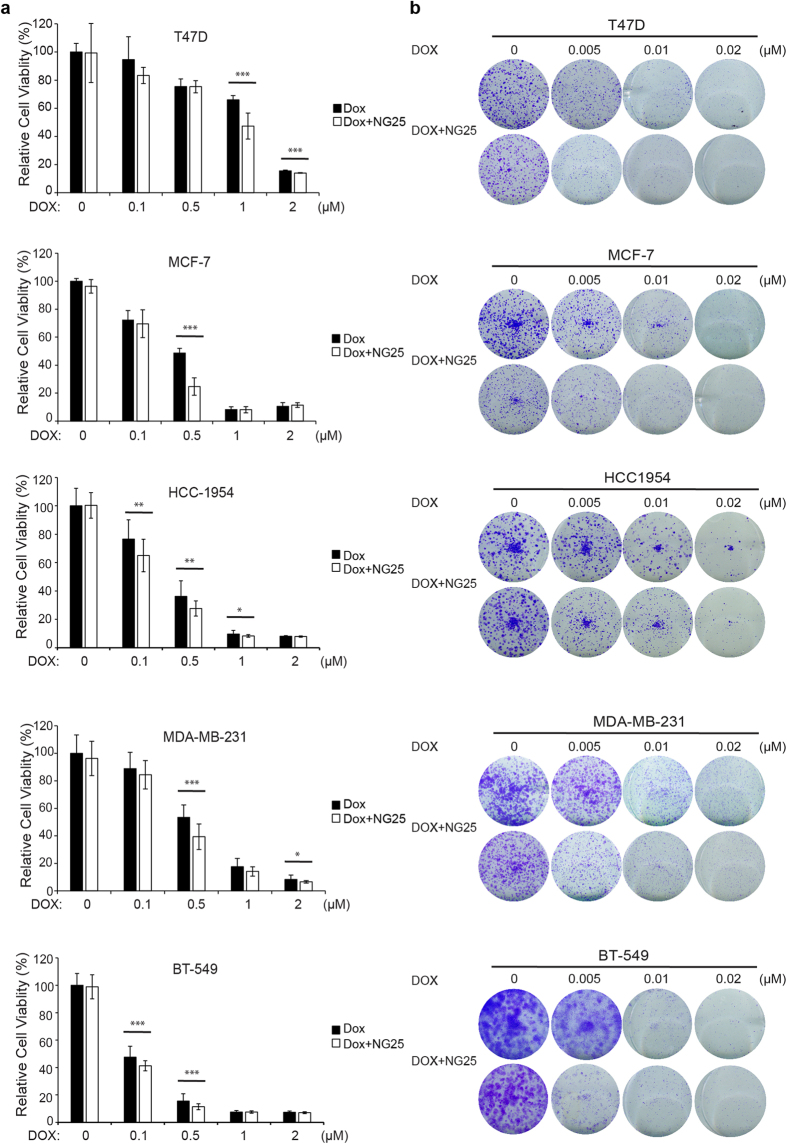
NG25 enhances the cytotoxic effect of Dox on breast cancer cells. (**a**) Breast cancer cell lines T-47D, MCF7, HCC1954, MDA-MB-231, and BT-549 were treated with Dox at the indicated concentrations with or without NG25 2 μM for 48 h. The cell viability was then measured by CCK-8 assay. Data were represented as mean ± SD. *P* values < 0.05 (*), < 0.01 (**), or < 0.001 (***) were indicated. (**b**) Five breast cancer cell lines were seeded in 12-well plates at 2 × 10^3^ per well, and then incubated with Dox at the indicated concentrations with or without 2 μM NG25 for 72 h and were cultured in fresh medium without drug. Then the cell colonies were fixed, stained with methanol/crystal violet dye, and photographed.

Because of the development of chemoresistance, monotherapies are rarely effective enough in the treatment of breast cancer. We evaluated whether NG25 could enhance the cytotoxic effects of Dox on the proliferation of the above five types of breast cancer cell lines. The cells were cultured in the increased concentration of Dox alone or in combination with 2 μM of NG25 for 48 h, and the cell proliferation was assessed by CCK-8 assay. The cell viabilities were lower when the cells were treated with the combination compared to that with Dox single agent treatment (Fig. 2a). It indicates that NG25 may sensitize the breast cancer cells to Dox-mediated cytotoxicity.

To further validate the combination effect of NG25 and Dox on cell proliferation, the cell colony formation assay was performed. The cells were cultured in the increased concentration of Dox alone or in combination with 2 μM of NG25 for 72 h and then in fresh medium without drug for two weeks. Dox and NG25 combination treatment showed stronger inhibitory effect on the cell proliferation comparing to Dox single treatment (Fig. 2b). These data imply that NG25 can enhance the inhibitory effect of Dox on breast cancer proliferation regardless of molecular subtypes.

### NG25 enhances the inhibitory effects of Dox on anchorage-independent growth of breast cancer cells

**Figure 3:**
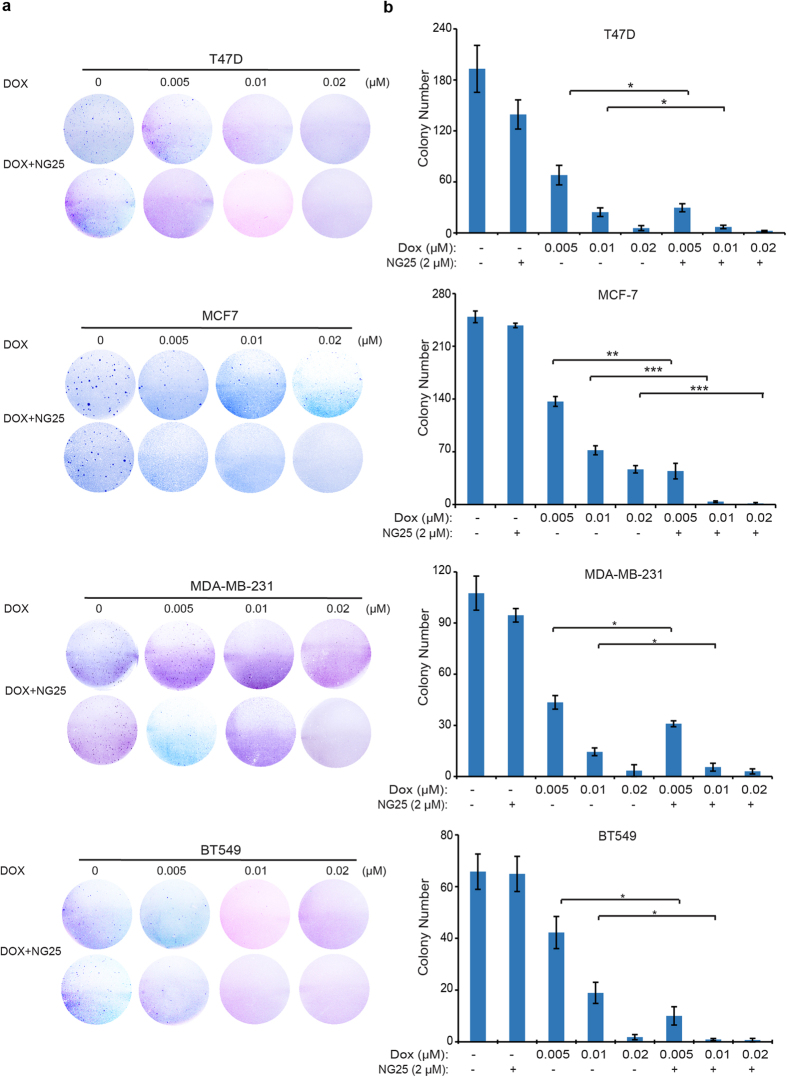
NG25 enhances the inhibitory effect of Dox on breast cancer Anchorage-independent growth. (**a**) Cell anchorage-independent growth ability was assessed by soft agar assay. Four breast cancer cell lines, T-47D, MCF7, MDA-MB-231, and BT-549 were incubated with Dox at the indicated concentrations with or without 2 μM NG25 in soft agar plates for three weeks, followed by staining with crystal violet dye and photographed. (**b**) The colonies of (**a**) were counted and colony numbers were represented as mean ± SD. *P* values < 0.05 (*), < 0.01 (**), or < 0.001 (***) were indicated.

Cancer cells maintain a special quality that they are able to grow colonies when cultured in soft agar. To assess whether NG25 could enhance the inhibitory effects of Dox in reducing the anchorage-independent growth ability of breast cancer cells, soft agar assays were performed. Breast cancer cells, including T-47D, MCF7, HCC1954, MDA-MB-231, and BT-549, were cultured with Dox alone at 0 μM, 0.005 μM, 0.01 μM and 0.02 μM or in combination with 2 μM of NG25 for three weeks. Then the visible colonies were fixed and stained. Because HCC1954 did not form visible colonies in this assay, the data of this cell is not shown. For the rest cell lines, the numbers of the colony decreased more obviously in Dox and NG25 combination treatment groups compared to the Dox single agent treatment groups (Fig. 3a). The differences were statistically significant in the cells tested (Fig. 3b). In conclusion, co-culture of breast cancer cells with Dox and TAK1 inhibitor NG25 significantly increased the sensitivity of breast cancer cells to Dox-mediated inhibitory effect.

### TAK1 inhibition suppresses Dox-induced p38 activation and IκBα degradation

**Figure 4:**
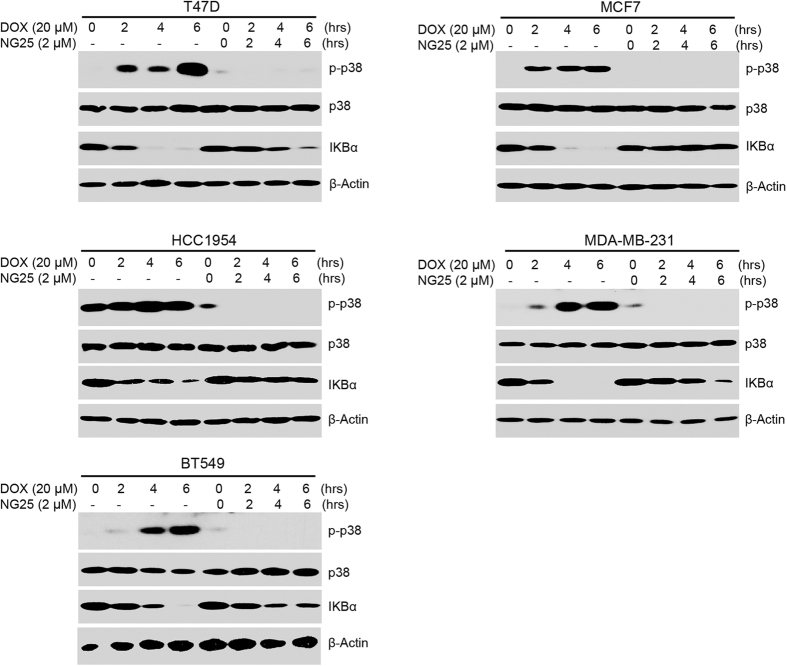
TAK1 inhibition inhibits Dox-induced p38 activation and IκBα degradation. Breast cancer cell lines T-47D, MCF7, HCC1954, MDA-MB-231, and BT-549 were treated with Dox (20 μM) alone or combined with NG25 (2 μM) for 0, 2 h, 4 h or 6 h. The protein extracts were subjected to SDS-PAGE and immunoblotted with the antibodies against p-p38, p38, and IκBα. β-actin were detected as loading controls for whole cell extracts.

NF-κB pathway is one of the key molecular pathways in regulating cell survival^15^. Studies suggested that Dox could induce NF-κB activation^19^. TAK1 is required for genotoxic stress-induced NF-κB and p38 activation^20^. To clarify this concept in breast cancer cells, we evaluated the effects of TAK1 inhibition with Dox mediated NF-κB activation in breast cancer cells. T-47D, MCF7, HCC1954, MDA-MB-231 and BT-549 cells were cultured with Dox alone or in combination with 2 μM NG25 for 2 h, 4 h, or 6 h, respectively. As shown in Fig. 4, TAK1 inhibition by NG25 blocked Dox-induced p38 phosphorylation and Dox-induced IκBα degradation. These data signify that TAK1 is required for Dox induced NF-κB and p38 activation in breast cancer cells, and NG25 can block Dox induced NF-κB and p38 activation in breast cancer cells.

### NG25 strengthens Dox-induced apoptosis in breast cancer cells

**Figure 5:**
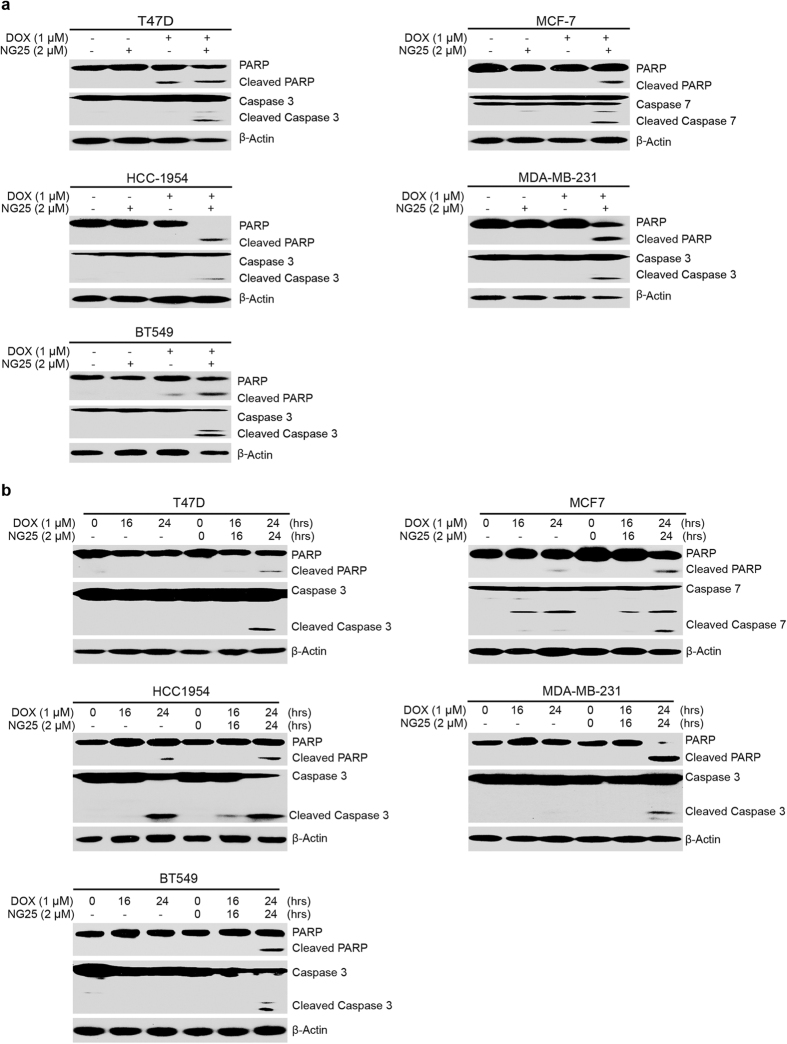
NG25 strengthens Dox-induced apoptosis in breast cancer cells. (**a**) Breast cancer cell lines T-47D, MCF7, HCC1954, MDA-MB-231, and BT-549 were treated with Dox (1 μM) alone or combined with NG25 (2 μM) for 24 h. Then the protein extracts subjected to SDS-PAGE and immunoblotted with the antibodies against PARP and Caspase 3 or Caspase 7 to detect the cellular apoptosis. β-actin were detected as loading controls. (**b**) Breast cancer cell lines T-47D, MCF7, HCC1954, MDA-MB-231, and BT-549 were treated with Dox (1 μM) alone or combined with NG25 (2 μM) for 0, 16 h, or 24 h. The protein extracts were subjected to SDS-PAGE and immunoblotted with the antibodies against PARP and Caspase 3 or Caspase 7 to detect the cellular apoptosis. β-actin were detected as loading controls for whole cell extracts.

To further examine the cytotoxic potency of NG25 in breast cancer, we tested whether NG25 could induce PARP and caspase-3 cleavages. Our immunoblotting results showed that NG25 alone could not induce PARP and Caspase-3 cleavages; but it significantly increased Dox-induced PARP and Caspase 3 or Caspase 7 cleavages (Fig. 5a). To further explore whether NG25 could enhance Dox-induced apoptosis in breast cancer cells, the cells were stimulated with Dox alone (1 μM) or combined with NG25 at 2 μM (lower dose) for 0, 16 h, or 24 h. Immunoblotting analyses demonstrated that NG25 could significantly enhance Dox-induced PARP and Caspase 3 or Caspase 7 cleavages in all subtypes of breast cancer cell lines tested (Fig. 5b). Taken together, these results indicate that NG25 could sensitize breast cancer cells to Dox-induced cellular apoptosis.

## Discussion

TAK1, as an intracellular hub molecule that regulates both NF-κB and MAPK signaling pathways, plays an important role in cell survival, promotion of metastasis and development of drug resistance^15^. Previous studies suggest that TAK1 may be a promising target for cancer treatment^4^. Inhibition of TAK1 by RNAi-silencing or an oral inhibitor LYTAK1 significantly enhanced the cytotoxic effect of oxaliplain and gemcitabine in pancreatic cancer^17^. TAK1 is a treatment target for enhanced efficacy of topoisomerase inhibitors in breast cancer^4^. Additionally, 5Z-O, a TAK1 inhibitor isolated from fungi enhanced the cell-killing activity of Dox and etoposide in neuroblastoma^21^. Furthermore, TAK1 inhibition had pro-apoptotic activity in KRAS-dependent colon cancer^18^. Recently, MicroRNA-26b has been shown to suppress the activation of NF-κB signaling and to enhance the chemosensitivity of hepatocellular carcinoma cells by targeting TAK1 and TAB3^29^. Consistent with these findings, we found that NG25, a synthesized TAK1 inhibitor, could sensitize the breast cancer cells to Dox mediated cytotoxicity through inhibiting the activation of NF-κB pathway. Herein, in this study, by using a panel of breast cancer cell lines, representing different molecular subtypes of breast cancer, we provide clear evidence that NG25 can enhance Dox-induced cytotoxicity in breast cancer cells through TAK1 inhibition.

The role of NF-κB as a master transcriptional factor promoting cell-survival, increasing therapeutic resistance and enhancing metastasis ability of cancer cells has been well documented^30^,^31^. NF-κB can be activated by multiple stimuli including genotoxic stresses^19^,^20^. Dox, as a widely used chemotherapy reagent in different types of cancer, triggers the apoptosis of cancer cells by interfering the DNA topoisomerase ‖α and creating DNA double-stand breaks which subsequently activates NF-κB signaling pathway in order to repair the damage via a stimulatory action on homologous repair, involving the targets ATM and the tumor suppressor gene, breast cancer susceptibility gene 2 (BRCA2)^7^,^32^. In our studies, NG25 is shown to inhibit Dox-induced NF-κB activation and disturb the balance between pro-apoptotic and pro-survival pathways and thus it increased the efficacy of Dox chemotherapy in breast cancer cells. By detecting the level of IκBα, we showed that NG25 can inhibit TAK1-mediated NF-κB activation, which possibly is one of the major mechanisms of NG25 activity in the breast cancer cells. Besides that, we also detected that the inhibition of TAK1-mediated MAPK activation had partially been blocked by NG25, which may also contribute to the effect of this drug on breast cancer cells. Taken together, we conclude that inhibition of TAK1 activity by NG25 can enhance Dox-induced apoptosis by blocking NF-κB and MAPK signaling pathways. Integration of NG25 with Dox chemotherapy can reduce Dox dosage and thus minimize its side effects.

As known, breast cancer is a heterogeneous disease, which can be classified into four major subtypes, including luminal A, luminal B, HER2 positive, and triple negative tumors. Each subtype has specific molecular changes, yet it is still possible to highlight common molecular features among those different tumor subtypes. Constitutively activation of NF-κB, a transcription factor that plays critical role in cell survival and proliferation, is one of those common features found in most breast cancer tumors^15^. Furthermore, TAK1 has been shown to be constitutively activated in human breast cancer by detecting the phospho-TAK1 level on tumor tissues^22^. Inhibition of TAK1 by RNAi knockdown of TAB1 significantly suppressed tumor growth and metastasis *in vivo*, suggesting that TAK1 is a potential therapeutic target for breast cancer. In our study, we found that NG25 alone has cytotoxic effect on all of the breast cancer cell lines in a dose-dependent manner (Fig. 1), but with varied efficacy. To be noted, TAK1 has been shown to be constitutively activated in highly metastatic MDA-MB-231 breast cancer cells while its activity is very low in non-metastatic MCF-7 cells^33^. Consistent with these results, we found that NG25 had a relatively stronger inhibitory effect on MDA-MB-231 cells compared with that on MCF-7 cells, suggesting that TAK1 may be a promising therapeutic target, especially for highly aggressive triple negative breast cancers (Fig. 1).

Targeting TAK1 as a strategy to overcome chemotherapy resistance has been reported in pancreatic cancer, neuroblastoma and hepatocellular carcinoma^17^,^21^,^29^. It has also been tested to reverse radiotherapy resistance in breast cancer^34^. In this study, we provide clear evidence that NG25 enhanced the Dox-mediated cytotoxic activity in different breast cancer cell lines. This activity seems universal regardless of the molecular subtypes of breast cancer cells. Therefore, targeting TAK1 may be an option to overcome the chemotherapy resistance.

Taken together, we demonstrated that TAK1 inhibitor NG25 can suppress the Dox-induced NF-κB and MAPK signaling pathway activation, which subsequently sensitized breast cancer cells to Dox-mediated cytotoxic effect and this effect is observed in all major breast cancer cell subtypes. The data we presented here suggest that NG25, as a potent inhibitor of TAK1-mediated NF-κB activation, might be useful in enhancing the efficacy of Dox while reducing its side effects and then broaden its clinical use, deserves further investigation and clinical validation.

## Materials and Methods

### Cell lines and cell culture

Human breast cancer cell lines T-47D, MCF7, HCC1954, MDA-MB-231, and BT-549 were purchased from American Type Culture Collection (ATCC, Manassas, VA, USA). Normal breast epithelial cell lines HMEC and MCF-12A were kindly provided by Dr. Shiaw-Yih Lin (University of Texas MD Anderson Cancer Center). MCF7 and MDA-MB-231 cells were grown in Dulbecco’s modified Eagle’s medium (DMEM, Lonza, Walkersville, MD, USA) containing 10% fetal bovine serum (FBS, Sigma-Aldrich Co. LLC. St. Louis, MO, USA), 100 units/ml penicillin, and 100 mg/ml streptomycin. T-47D, HCC1954 and BT-549 were maintained in RPMI-1640 medium (Lonza), containing 10% FBS, 100 units/ml penicillin, and 100 mg/ml streptomycin. HMEC and MCF-12A cells were grown in MEBM supplemented with MEGM Single Quots and cholera toxin (Lonza). All cells were grown at 37 °C in a humidified atmosphere of 5% CO_2_.

### Antibodies and reagents

TAK1 inhibitor NG25 (HY-15434) was purchased from MedChem Express (Monmouth Junction, NJ, USA) and was prepared according to the manufacturer’s recommendations. The antibodies against PARP (9532), Caspase 3 (9662), Caspase 7 (12827), IκBα (9242), phospho-p38 MAP kinase (Thr180/Tyr182) (9211), p38 MAP kinase (8690), anti-Mouse (7076S) and anti-Rabbit (7074S) IgG were purchased from Cell Signaling Technology (Danvers, MA, USA). Doxorubicin (Dox, D1515) and antibody against β-actin (A2228) were obtained from Sigma-Aldrich Corp (St. Louis, MO, USA).

### Cell Viability assay

Cell viability assays were performed using the CCK-8 from Dojindo Laboratories (Rockville, MA, USA) following the manufacturer’s instructions. Briefly, cells were seeded and grown in 96-well clear-bottom plates starting at 5 × 10^3^ cells per well. After 24 h of incubation, cells were either grown in media alone or media containing increasing concentrations of NG25, Dox, or the combination of NG25 and Dox for 48 h or 72 h. Then cell morphologies were observed and captured using an optical microscope. After observation, a mixture of 10 μl of CCK-8 and 190 μl media was added into each well and the cells were incubated for another 1 h. The absorbance of each well was measured at 450 nm using a microplate reader. Each experiment was performed in triplicates.

### Colony formation assay

Breast cancer cells were seeded in 12-well plates at 2 × 10^3^ cells per well, then incubated with Dox alone or Dox with NG25 at indicated concentrations after 72 h and then were cultured in fresh medium without drug. Two weeks later, cells were fixed and stained with methanol/crystal violet for 10 min, and photographed. Each experiment was performed in triplicates.

### Anchorage-independent growth assay

Cell anchorage-independent growth ability was assessed by soft agar assay. As previously described, a 5%(w/v) base agar (214220, Difco Laboratories, Detroit, MI, USA) was made by adding the agar into distilled water and autoclaved. Then the solution was allowed to cool in a 56 °C water bath. After that, the lower 0.5% agar, which contains RPMI or DMEM and 12% FBS, was added into 6-well plates (2 mL per well). For the top layer, each breast cancer cell line was mixed with 0.3% agar 1.5 ml in each well at the density of 5 × 10^3^ cells along with indicated concentrations of NG25, Dox or their combination respectively. Cells were grown at 37 °C for 3 weeks until the colonies were visible to the naked eye, then stained with crystal violet for 2 h and photographed. The colonies were counted by Quantity One software (Bio-Rad Laboratories, Inc., Hercules, CA, USA) and plotted. Each experiment was performed in triplicates.

### Immunoblotting

For immunoblotting, Cell lysates were obtained by washing the cells twice with ice cold PBS, and spun down. The cell pellets were dissolved in lysis buffer on a rotator in 4 °C for 30 min [50 mM Tris-HCl at pH 7.4, 150 mM NaCl, 1 mM EDTA, 1% NP-40, 0.25% sodium deoxycholate, 1 mM phenylmethylsulfonyl fluoride (PMSF), 1 mM benzamidine, 10 μg/mL leupeptin, 1 mM dithiothreitol (DTT), 10 mM sodium fluoride (NaF), 0.1 mM sodium orthovanadate (OV), phosphatase inhibitor cocktail 2 and 3 (p5726 and p0044, Sigma-Aldrich)]. After centrifuging at 13,000 rpm for 15 min, the supernatants were collected as cell lysates. Protein concentrations were measured using Bradford reagent (Bio-Rad Laboratories, Hercules, CA, USA). After mixed with 4 × loading buffer and heated to 100 °C for 6 min, cell lysates were subjected to 10% or 15% SDS–PAGE electrophoresis and transferred to PVDF membranes, followed by blocking with 5% milk for 1 h at room temperature (25 °C). Then the PVDF membranes was incubated with corresponding primary antibodies at 4 °C overnight and the horseradish peroxidase-conjugated antibodies against rabbit or mouse IgG at RT (25 °C) for 1 h. The membranes were developed using the ECL Western blotting system (Thermo Fisher Scientific Inc., Rockford, IL, USA) according to the manufacturer’s instruction.

### Statistical analysis

All values were presented as mean ± standard deviation (SD). *P < 0.05, **P < 0.01, ***P < 0.001 were considered to be statistically significant. Student’s *t*-test (two-tailed) was used to analyze the difference between Dox treatment groups and combination treatment group.

## Additional Information

**How to cite this article**: Wang, Z. *et al*. TAK1 inhibitor NG25 enhances doxorubicin-mediated apoptosis in breast cancer cells. *Sci. Rep.* **6**, 32737; doi: 10.1038/srep32737 (2016).

## Supplementary Material

Supplementary Information

## Figures and Tables

**Table 1:** Molecular classification of the human breast cancer cell lines^27^,^28^.

	Cell line	Classification	ER	PR	HER2	Response to Chemotherapy
1	T-47D	Luminal A	+	+	−	Responsive
2	MCF7	Luminal A	+	+	−	Responsive
3	HCC1954	HER2	−	−	+	NA
4	MDA-MB-231	Claudin-low	−	−	−	Intermediate
5	BT-549	Claudin-low	−	−	−	Intermediate

ER, estrogen receptor; PR, progesterone receptor; HER2, human epidermal growth factor receptor 2; NA, not available.
