# Supplementary material for: TAK1 inhibitor NG25 enhances doxorubicin-mediated apoptosis in breast cancer cells
Source: Sci Rep. 2016 Sep 7;6:32737. doi: 10.1038/srep32737 (PMC5013439; doi:10.1038/srep32737)
Supplement: Supplementary Information [file srep32737-s1.pdf]

TAK1 inhibitor NG25 enhances doxorubicin-mediated apoptosis in breast cancer cells

Zhenyu Wang[1, 2,3], Huiyuan Zhang[2], Minghao Shi[2.4], Yang Yu[2], Hao Wang[2,5], Wen-Ming Cao[2,6], Yanling Zhao[2], Hong Zhang[2,3,*]

[1]Department of Breast Surgery, the Second Hospital of Jilin University, Changchun, Jilin 130041, China

[2]Department of Pathology, University of Texas MD Anderson Cancer Center, Houston, Texas 77030, USA

[3]Department of Translational and Molecular Pathology, University of Texas MD Anderson Cancer Center, Houston, Texas 77030, USA

[4]Department of Blood Transfusion, the Second Hospital of Jilin University, Changchun, Jilin 130041, China

[5]Department of Hepatopancreatobiliary Surgery, the Second Affiliated Hospital of Harbin Medical University, Harbin, Heilongjiang 150086, China.

[6]Department of Medical Oncology, Zhejiang Cancer Hospital, Hangzhou, Zhejiang 310022, China

Supplemental Figure S1

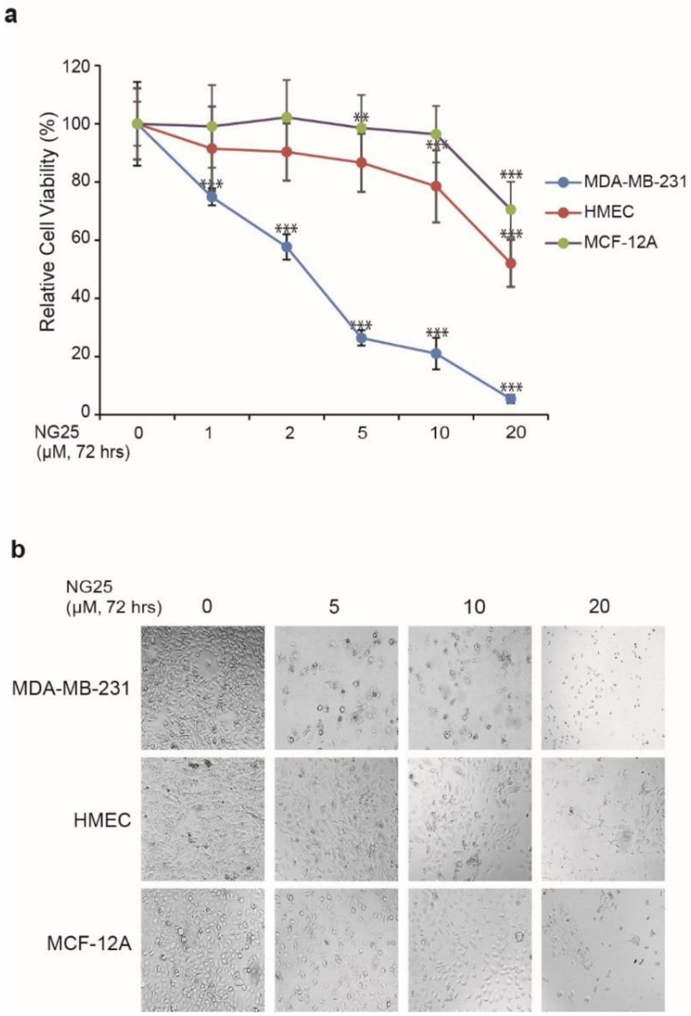

Supplemental Figure Legends

Supplemental Fig.S1 NG25 shows much lower toxicity to normal breast epithelial cell lines HMEC and MCF-12A compared to MDA-MB-231 cell line. (**a**) Two normal breast epithelial cell lines and MDA-MB-231 were treated with the indicated concentrations of NG25 for 72 hrs. Cell viability was then measured by adding the mixture of 10 μL of CCK-8 and 190 μL medium and reading the absorbance at 450 nm. Data were represented as mean ± SD. *P* values <0.01 (**) or <0.001 (***) were indicated. (**b**) Two normal breast epithelial cell lines and MDA-MB-231 were treated with the indicated concentrations of NG25 for 72 hrs, and cell morphology was captured using optical microscope.
